# Identification of Karyopherin α1 and α7 Interacting Proteins in Porcine Tissue

**DOI:** 10.1371/journal.pone.0038990

**Published:** 2012-06-13

**Authors:** Ki-Eun Park, H. Dorota Inerowicz, Xin Wang, Yanfang Li, Stephanie Koser, Ryan A. Cabot

**Affiliations:** 1 Department of Animal Sciences, Purdue University, West Lafayette, Indiana, United States of America; 2 Bindley Biosciences Center, Proteomics Core Facility, Purdue University, West Lafayette, Indiana, United States of America; University College Dublin, Ireland

## Abstract

Specialized trafficking systems in eukaryotic cells serve a critical role in partitioning intracellular proteins between the nucleus and cytoplasm. Cytoplasmic proteins (including chromatin remodeling enzymes and transcription factors) must gain access to the nucleus to exert their functions to properly program fundamental cellular events ranging from cell cycle progression to gene transcription. Knowing that nuclear import mediated by members of the karyopherin α family of transport receptors plays a critical role in regulating development and differentiation, we wanted to determine the identity of proteins that are trafficked by this karyopherin α pathway. To this end, we performed a GST pull-down assay using porcine orthologs of karyopherin α1 (KPNA1) and karyopherin α7 (KPNA7) and prey protein derived from porcine fibroblast cells and used a liquid chromatography and tandem mass spectrometry (LC-MS/MS) approach to determine the identity of KPNA1 and KPNA7 interacting proteins. Our screen revealed that the proteins that interact with KPNA1 and KPNA7 are generally nuclear proteins that possess nuclear localization signals. We further validated two candidate proteins from this screen and showed that they are able to be imported into the nucleus *in vivo* and also interact with members of the karyopherin α family of proteins *in vitro*. Our results also reveal the utility of using a GST pull-down approach coupled with LC-MS/MS to screen for protein interaction partners in a non-traditional model system.

## Introduction

Chromatin remodeling (whether mediated by histone modifying enzymes, DNA methyltransferases, or nucleosome repositioning complexes) directly affects the ability of transcription factors to access chromatin, thus tightly linking these two fundamental cellular processes (chromatin remodeling and gene transcription) together [1]–[3]. In eukaryotic cells, the presence of the nuclear envelope provides a layer of separation between the cytoplasm, where transcription factors and chromatin remodeling enzymes are synthesized, and the nucleus and the chromatin contained therein. Several intracellular trafficking systems have been described [4], [5]; each uses a distinct mechanism to interact with nuclear cargoes (for export to the cytoplasm) and cytoplasmic cargoes (for import to the nucleus). These trafficking systems transport their intracellular cargoes through the nuclear pore complexes on the nuclear envelope and position the cargoes in the proper cellular compartment at the correct time during the cell cycle and development.

Nuclear import mediated by the karyopherin α/β heterodimer is perhaps the best biochemically characterized nuclear trafficking system in mammals [6]. Here, karyopherin α serves as an adaptor protein and interacts with a nuclear localization signal (NLS) found within the primary amino acid sequence of a cytoplasmic protein destined for the nucleus. Once bound to its NLS-bearing cargo, karyopherin α then binds to karyopherin β and this trimeric complex translocates across the nuclear envelope into the nucleus [7]. The NLSs recognized by karyopherin α vary in sequence identity but can generally be described as being monopartite or bipartite. Monopartite NLSs consist of a single cluster of basic amino acids (such as arginine and lysine residues), and are exemplified by the NLS within the SV40 large T-antigen (-PKKKRKV-). Bipartite NLSs consist of two shorter stretches of basic amino acids separated by a spacer of approximately 10 amino acids; bipartite NLSs are exemplified by the NLS found within nucleoplasmin (-KRPAATKKAGQAKKK-) [8].

Seven karyopherin α (KPNA) subtypes have been described in mammals [9]–[11]. Although these subtypes share the same general structure and have a high degree of sequence identity, data suggest that these subtypes are not functionally redundant. Firstly, porcine embryos appear to have differing developmental requirements for KPNA2 and KPNA3 during cleavage development [12]. Secondly, although all karyopherin α subtypes are able to interact with proteins bearing NLSs, there appears to be some substrate specificity with regard to how strongly individual karyopherin α subtypes interact with a given NLS-bearing substrate [13]. For example, bovine KPNA7 appears to have a considerably higher binding affinity for nucleoplasmin 2 than other karyopherin α subtypes [9]. In addition, the Ran GTP/GDP nucleotide exchange factor (RCC1) and Ran binding protein 3 (RanBP3) are preferentially trafficked by importin α3 (KPNA4) [14], [15]. The members of the karyopherin α family have also been shown to be differentially expressed in various adult tissues. In the testis for example, karyopherins α3 and α4 have been detected in the Leydig cells, while proteins in the karyopherins α1, α5 and α6 have been found in all cells of the seminiferous tubules [16]; karyopherin α subtypes have been shown to be differentially expressed in the developing male germ cells as they proceed through spermatogenesis [17].

Nuclear trafficking mediated by karyopherin α has also been shown to play a key role during mammalian embryo development and cellular differentiation. The murine knockouts for KPNA6 (also known as importin α7) and KPNA1 (also known as importin α5) show drastically different phenotypes. KPNA6 null females produce oocytes that fail to develop beyond the stage of zygotic genome activation upon fertilization [18], while KPNA1 knockout animals appear phenotypically normal [19]. Bovine and porcine embryos appear to require KPNA7 to complete cleavage development [9], [11]. Differentiation of murine embryonic stem (ES) cells has been shown to be modulated by altering levels of specific karyopherin α subtypes [20]. Together these data strongly suggest that unique cargoes are trafficked by the individual members of the karyopherin α family, and that karyopherin α/β-mediated nuclear trafficking may function as a regulatory switch during development and differentiation.

Here we report the use of a liquid chromatography-mass spectrometry (LCMS) approach coupled with a GST pull-down assay to determine the identity of intracellular proteins that interact with the porcine orthologs of KPNA1 and KPNA7. These karyopherin alpha subtypes were chosen based on the fact that they show differential tissue-specific expression, have been shown to interact with differing affinities toward different NLS-bearing cargoes [9], [11]. We find that the majority of proteins identified by our screen either possess a putative NLS or are known nuclear proteins. In addition, we could validate our findings by using both a microinjection assay and an *in vitro* binding assay to show that candidate proteins can be trafficked to the nucleus *in vivo* and physically interact with karyopherin α proteins *in vitro*. This work demonstrates the power of using a pull-down assay coupled with LCMS analysis to screen for protein interacting partners in a non-traditional model system.

## Materials and Methods

### Ethics Statement

All experiments involving oocytes and embryos were performed with the specific approval of the Purdue Animal Care and Use Committee.

### Vector Construction

Gateway cloning technology (Invitrogen) was used to generate green fluorescent protein (GFP) and glutathione-S-transferase (GST) tagged versions of the following porcine karyopherin α (KPNA) proteins: KPNA1, KPNA2, KPNA3, KPNA5, and KPNA7 (Genbank accession numbers GQ166955, GQ166956, GQ166957, XM_001925212, and GQ166958). Briefly, the open reading frames of the porcine orthologs of KPNA1, KPNA2, KPNA3, KPNA5, and KPNA7 were amplified by PCR and cloned into the pENTR/SD/D-TOPO vector (Invitrogen) and subsequently recombined into the pDEST53 vector (Invitrogen) to generate the GFP expression constructs. A partial open reading frame of porcine KPNA6 (Genbank accession number XM_003356277) was also cloned as described above; however, this particular clone was truncated at the 5′ end and was missing part of the importin β binding domain, and possessed only amino acids 46–533. All karyopherin α constructs present in the pENTR/SD/D-TOPO vectors were also recombined into the pDEST15 vector (Invitrogen) to generate the GST expression constructs. An open reading frame that encompassed the presumptive NLS found within porcine sperm associated antigen 17-like protein (SP17) was amplified by PCR from cDNA synthesized from porcine fetal fibroblast mRNA with primers spanning positions 1660–2289 within accession number XM_001927306; the resultant PCR product was cloned into the pENTR/SD/D-TOPO vector and subsequently recombined into the pDEST15 vector to generate a GST expression construct. An open reading frame that encompassed the presumptive NLS found within porcine ras responsive element binding protein 1, transcript variant 1(RREB) was amplified by PCR from cDNA synthesized from porcine fetal fibroblast mRNA with primers spanning positions 1–453 within accession number XM_001927306; the resultant PCR product was cloned into the pENTR/SD/D-TOPO vector and subsequently recombined into the pDEST15 vector to generate a GST expression construct. The open reading frame of porcine karyopherin β (XM_003131528) was amplified by PCR from cDNA synthesized from porcine fetal fibroblast mRNA and cloned into the pENTR/SD/D-TOPO vector and subsequently recombined into the pDEST15 vector to generate a GST tagged version of porcine karyopherin β.

### Protein Production and Fluorescent Labeling of SP17, RREB, NLS and GST

BL21 cells were transfected with the DNA vectors coding for GST fusion proteins containing GST-KPNA1, GST-KPNA7 and GST-alone for use in our GST pull-down assay; GST fused with either the SV-40 T-antigen NLS (NLS) [11], SP17, or RREB, and GST alone were produced for use in our *in vitro* binding and microinjection assays. Cells were grown in liquid culture medium to an OD_600_ ranging between 0.5–0.8 at 37°C; protein expression was then induced with 0.2 mM IPTG and 0.2% arabinose at 25°C for 5 hours. Following incubation, liquid cultures were cooled to 4°C and the bacteria were pelleted by centrifugation. Bacterial pellets were resuspended in PBS containing 0.3% Triton X-100, 1 mM dithiothreitol, and protease inhibitors (Complete Protease Inhibitor Cocktail tablets, catalog number 04-693-159-001, Roche, Indianapolis, IN) and lysed by sonication. After centrifugation at 12,000 xg for 10 minutes, the cleared bacterial lysates were applied to glutathione agarose beads according to the manufacturer’s instructions (Sigma). For fluorescent labeling of proteins, following three washes with cold PBS, but before elution from the beads, GST alone, RREB and SP17 proteins were labeled with fluorescein-5-maleimide (Pierce, Rockford, IL) according to the manufacturer’s protocol. GST-NLS was labeled with Alex 594 (Invitrogen) according to the manufacturer’s protocol. Following labeling, the beads were washed three additional times with cold PBS to remove free, unbound label. Labeled GST proteins were eluted from the beads with a cold solution of 10 mM reduced glutathione in 50 mM Tris-HCl pH 8.0. Protein solutions were divided into 10 µl aliquots and stored at -80°C.

### GST pull-down Assay

A total protein lysate produced from porcine fibroblast cells derived from a day 45 fetus at the fourth passage was used as the prey protein in the GST pull-down assay. Here, the fibroblasts were grown to confluency in DMEM containing 15% fetal bovine serum in a 31 cm^2^ culture dish. The confluent culture was washed twice in DPBS and then coated with 1 ml of M-PER (Thermo-Scientific, catalog number 78503); the culture was held at 4°C for 2 hours with constant rotation. The suspension was then centrifuged at 12,000 x g for 10 minutes and the cleared supernatant removed for further processing. Protein concentration was determined by the Bradford assay and aliquots stored at -80°C. The prey protein (10 mg) was then co-incubated with glutathione agarose beads (1 ml bead slurry plus 9 ml PBS); beads were removed after co-incubation for 2 hours at 4°C by centrifugation at 10,000 x g for 1 minute to yield cleaned prey protein. This step served to reduce non-specific binding of prey proteins to the purification matrix. Bacterial lysates containing GST, GST-KPNA1 and GST-KPNA7 were incubated with 200 µl of glutathione agarose beads in a 1.5 ml microcentrifuge tube; following a 2 hour incubation at 4°C, beads were washed three times with PBS containing 0.3% Tween-20 (PBST), followed by three washes with PBS. Following washes, 1 ml of the clean prey protein (e.g., 1 mg) was added to bound GST proteins and co-incubated for 2 hours at 4°C. Beads were washed three times with PBST, followed by three washes with PBS; proteins were eluted in 100 µl Laemmli sample buffer (Bio-Rad), boiled for 5 minutes and loaded into a 10% TGX precast gel (Bio-Rad). To determine the binding to KPNA1 and KPNA7 proteins (protein-protein interaction), gels were silver stained using the Pierce Silver Staining kit (catalog number 24612, Thermo-Scientific, ) according to the manufacturer’s instructions.

### Proteomic Analysis

Differential bands visualized in the KPNA1+prey and KPNA7+prey lanes of the silver stained gels were excised and processed by the Purdue Proteomics core facility in the Bindley Biosciences Center for in-gel extraction [28] and LC-MS/MS analysis. Briefly, the excised gel containing protein bands of interest was washed 4 times in a solution of 50% acetonitrile, 25 mM NH_4_HCO_3_ and then dried in a speedvac for 30 minutes. The sample were reduced with 10 mM dithiothreitol (DDT) for 1 h at 55°C and cysteine residues were alkylated with 55 mM iodoacetamide at room temperature. The samples were then digested overnight at 37°C using sequencing grade trypsin (Promega Corporation, Madison, WI). Following digestion, the peptides were extracted from gel pieces by sonication in 60% acetonitrile and 5% trifluoroacetic acid for 15 minutes at 4°C. Cleared supernatants were transferred to fresh siliconized tubes and dried in the speedvac. The samples were reconstituted in 5% acetonitrile/94.9% water/0.1%formic acid and separated in a nano-HPLC system (Agilent 110, Agilent Technologies, San Jose, CA). Tryptic peptides were loaded on the Agilent Zorbax 300SB -C18 enrichment column and incubated for 5 minutes. The enrichment column was switched into nano-flow path and peptides were separated with a Zorbax 300SB-C18 reversed phase analytical column (150 mm×75 µm, 3.5 µm) coupled to the electrospray ionization (ESI) nano-source of the high resolution hybrid ion trap mass spectrometer (LTQ-Orbitrap, Thermo Scientific). The peptides were eluted from the column with a linear gradient of 5–40% buffer B (acetonitrile/0.1% formic acid) in buffer A (water/0.1% formic acid) over 35 minutes at a rate of 300 nl/minute followed by gradient of 40–90% over 10 minutes. The column was equilibrated with 5% acetonitrile, 0.1% formic acid buffer for 20 minutes. The HPLC system was controlled by Agilent ChemStation software. The MS spectra were acquired in positive mode in a range from 300–2000. The MS/MS spectra were acquired in a data dependent acquisition mode. The four most abundant ions in each MS spectrum were automatically selected for fragmentation. Dynamic exclusion parameters used: repeat counts (1), repeat duration 30 seconds, exclusion duration of 60 seconds. LC-MS/MS spectra were analyzed using Spectrum Mill software (Agilent Technologies) and searches were performed against the NCBI database. For LC-MS/MS peptide identification, only peptides with a Spectrum Mill score of 5 or higher and Spectrum Mill Scored Peak Intensity (SPI) of 50% or higher were considered positive.

### Oocyte Collection

All chemicals were obtained from Sigma Chemical Company (St. Louis, MO) unless stated otherwise. Prepubertal porcine (*Sus scrofa*) ovaries were donated by a local abattoir and transported to the laboratory in an insulated container. Cumulus-oocyte-complexes (COCs) were collected by manual aspiration of antral ovarian follicles 3–6 mm in diameter. Follicular fluid was pooled and allowed to settle by gravity; COCs were resuspended in HEPES-buffered medium containing 0.01% polyvinyl alcohol (PVA) [21]. COCs with multiple layers of intact cumulus cells were selected for the experiments. For germinal vesicle (GV)-stage oocytes used in microinjection, COCs were vortexed in 0.1% hyaluronidase in HEPES-buffered medium for 7 minutes to remove the cumulus cells.

### *In vitro* Maturation, *In vitro* Fertilization and Embryo Culture

Fifty to 75 COCs were placed in 500 µl of tissue culture medium 199 containing 0.14% PVA, 10 ng/ml epidermal growth factor, 0.57 mM cysteine, 0.5 IU/ml porcine FSH, and 0.5 IU/ml ovine LH and matured for 42–44 hours at 39°C and 5% CO_2_ in air, 100% humidity [21]. COCs were vortexed in 0.1% hyaluronidase in HEPES-buffered medium containing 0.01% PVA for 4 minutes to remove the cumulus cells following maturation. Groups of 30–35 mature, denuded oocytes were placed in 100 µl of a modified Tris-buffered medium (mTBM) and fertilized according to an established protocol [22], using fresh, extended boar semen. Briefly, boar semen was extended in Modena Boar Semen Extender (Swine Genetics International, USA) and kept at 17.5°C for up to three days. Before fertilization, 1–2 ml of extended semen was mixed with Dulbecco’s Phosphate Buffered Saline containing 1 mg/ml BSA (DPBS) to a final volume of 10 ml and centrifuged at 1000 xg, 25°C, for four minutes; spermatozoa were washed in DPBS a total of three times. After the final wash, spermatozoa were resuspended in mTBM. The sperm suspension was added to oocytes at a final concentration of 5×10^5^ spermatozoa/ml; gametes were co-incubated for 5 hours at 39°C and 5% CO_2_. Presumptive zygotes were injected with fluorescently-labeled proteins and subsequently placed in Porcine Zygote Medium 3 (PZM3) supplemented with 3 mg/ml fatty acid-free BSA [23] and cultured at 39°C, 5% CO_2_ and 100% humidity.

### Microinjection and Localization Signal Imaging

Labeled GST proteins were injected into denuded GV-stage oocytes for experiments involving GV-stage oocytes and injected into presumptive pronuclear stage embryos (5 hours after gamete mixing) for experiments involving pronuclear, 2-cell, 4-cell and 8-cell stage embryos. GV-stage oocytes were incubated for 30 minutes, 3 hours and 6 hours following labeled protein injection in *in vitro* maturation medium at 39°C and 5% CO_2_ in air. Presumptive zygotes were incubated for 15, 30, 48 or 72 hours following microinjection to assess the intracellular localization of the labeled proteins in pronuclear, 2-cell, 4-cell and 8-cell stage embryos. Embryos and GV-stage oocytes were fixed at their respective stages in 3.7% paraformaldehyde at room temperature for 15 minutes. After three washes in HEPES-buffered medium, cells were stained with Hoechst 33342 (2 µg/ml) for 15 minutes, mounted on glass slides in Vectashield (Vector Laboratories, Inc., Burlingame, CA), covered with a glass coverslip and sealed with fingernail polish. The slides were examined using both an epifluorescent microscope (Leica DM-IRB, Buffalo Grove, IL, USA) and a Zeiss LSM 710 confocal microscope. Representative images were captured using both the Leica DM IRB inverted microscope equipped with epifluorescence and a Zeiss LSM 710 confocal microscope.

### *In vitro* Binding Assay

Methionine-[^35^S] radiolabeled versions of KPNA1, KPNA2, KPNA3, KPNA5, KPNA6, and KPNA7 proteins were produced in rabbit reticulocytes using TNT Quick Coupled Transcription/Translation System (Promega Corporation, Madison, WI, USA) according to the manufacturer’s protocol, using the above-described GFP-tagged karyopherin α vectors as *in vitro* transcription templates. These vectors have been previously used as templates for in vitro transcription/translation [11]. Binding reactions for each karyopherin α subtype were carried out by incubating the respective reticulocyte lysates with 10 µg of either GST, GST-NLS, GST-karyopherin β (GST-KPNB), GST-SP17, or GST-RREB. GST proteins, glutathione agarose beads, and reticulocyte lysates were co-incubated in binding buffer (50 mM Tris-HCl, 150 mM NaCl, 5 mM EGTA and 1% Triton X-100, pH 7.3) for 2 hours at 4°C. Glutathione agarose beads were then washed three times with PBS. Samples were then mixed with Laemmli loading buffer, boiled for 5 minutes and resolved on a 10% TGX precast gel (Bio-Rad). Gels were stained with Coomassie blue, then dried and exposed to film at -80°C for 24–48 hours. Binding reactions were performed such that each respective *in vitro* transcribed and translated karyopherin α subtype was divided among five binding reactions (GST, GST-SP17, GST-RREB, GST-NLS, GST-KPNB). In addition, gels were resolved as indicated in the corresponding figure for each binding assay, plus one lane containing the crude lysate from the respective S^35^-labeled KPNA *in vitro* translation reactions, corresponding to 10% of input into each binding reaction. The intensities of protein bands were analyzed with Molecular Imaging Software (Carestream Health, Woodbridge, CT). For KPNA1, KPNA2, KPNA3, KPNA5, and KPNA7, binding intensities were normalized to the intensity of the GST-KPNB reaction (100%); because the KPNA6 clone lacked the majority of the importin β binding domain, KPNA6 binding reactions were normalized to the lysate input (100%).

## Results

### Identification of Proteins that Interact with Porcine KPNA1 and KPNA7

**Figure 1:**
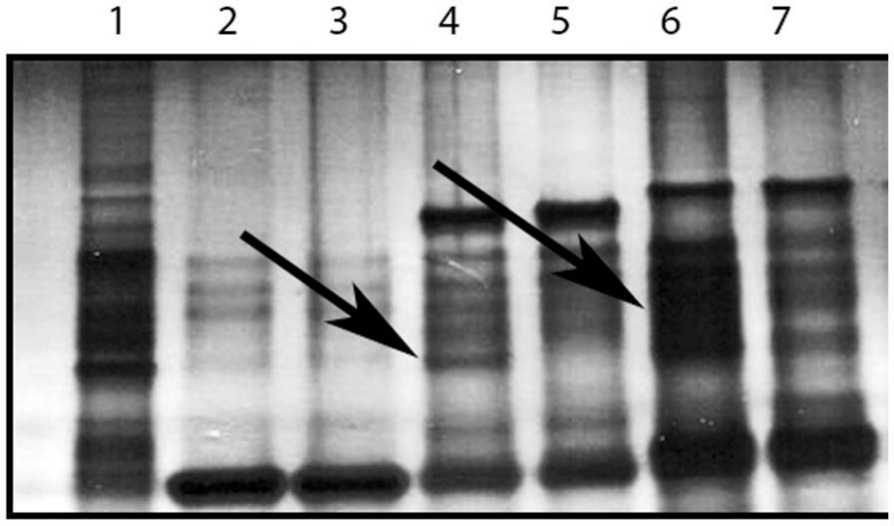
PAGE analysis of KPNA1 and KPNA7 GST pull-down reactions. A representative image of the resolved protein eluate following the GST pull-down assay. Lanes are labeled as follows: Lane 1, cleaned prey protein input (not eluted from GST column); Lane 2, GST and prey protein; Lane 3, GST alone; Lane 4, GST-KPNA7 and prey; Lane 5, GST-KPNA7 alone; Lane 6, GST-KPNA1 and prey; Lane 7, KPNA1 alone. Arrows indicate location of bands that were excised for proteomic analysis.

A GST pull-down assay was performed in which GST fusions of porcine KPNA1 and KPNA7, and GST alone were used as bait proteins to screen a prey protein source composed of total cellular protein derived from cultured porcine fetal fibroblast cells. Figure 1 shows the silver stained banding patterns from the respective binding assays. Bands that were unique to the KPNA1+prey and KPNA7+prey reactions were excised and subjected to tandem LC-MS/MS analysis to determine the identity of the proteins located in the differential bands. Table 1 shows the proteins identified by this LC-MS/MS analysis. A total of seven unique proteins were identified in the GST-KPNA1 pull-down; of the four proteins not attributed to background contamination (i.e., keratin and trypsin proteins), three were found to possess a putative NLS based on sequence analysis (https://www.predictprotein.org/) or were reported to be associated with the nucleus (www.uniprot.org). A total of eight unique proteins were identified in the GST-KPNA7 pull-down; of the five proteins not attributed to background contamination (i.e., keratin and trypsin proteins), four were found to possess a putative NLS based on sequence analysis (https://www.predictprotein.org/) or were reported to be associated with the nucleus (www.uniprot.org).

**Table 1:** KPNA1 and KPNA7-interacting proteins identified from porcine fibroblast cell lysate.

KPNA1 analysis				
% Scored Peak Intensity	Accession number	Protein name	Putative NLS	Intracellular localization
100	136429	RecName: Full=Trypsin	no	n/a
92.9	146741296	keratin 1	no	n/a
97	148747492	keratin 2	no	n/a
76	194041959	Polycomb group RING fingerprotein 6	no	nuclear
72.4	194044164	Vacuolar protein sorting 37homolog A	no	nuclear
69.9	194040638	hypothetical protein	no	unknown
76.7	194036373	sperm associated antigen 17	yes	cytoplasmic
**KPNA7 analysis**				
**% Scored Peak Intensity**	**Accession number**	**Protein name**	**Putative NLS**	**Intracellular localization**
98.3	136429	RecName: Full=Trypsin	no	n/a
95.2	146741296	keratin 1	no	n/a
85.5	148747492	keratin 2	no	n/a
87	140083661	ubiquitin B	no	nuclear
81.2	194037963	ras responsive element bindingprotein 1 isoform 1	yes	nuclear
79.9	4590045	glutamate receptor ionotropicAMPA 1 precursor	no	membrane bound
74.3	194041959	Polycomb group RING fingerprotein 6	no	nuclear
74.9	194034409	Fanconi anemia, complementationgroup M	yes	nuclear

### SP17 and RREB are Imported into the Nuclei in Cleavage Stage Embryos

**Figure 2:**
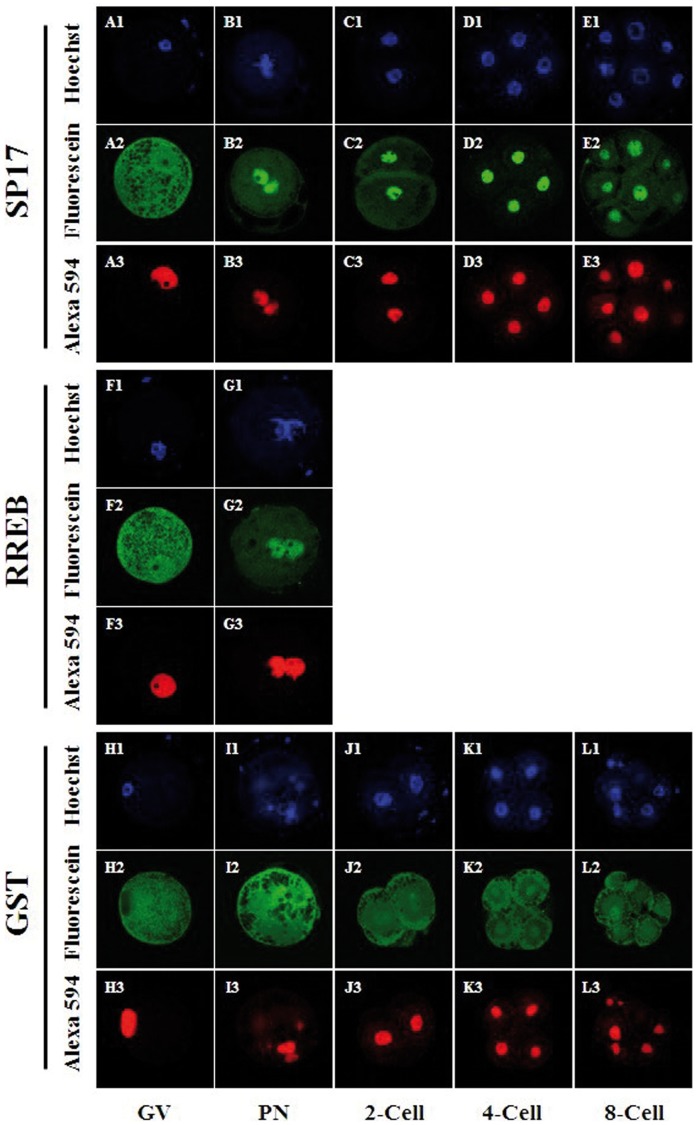
Recombinant GST-SP17 and GST-RREB adopt a nuclear localization. Fluorescein-labeled GST-SP17 and GST-RREB adopt a nuclear localization in porcine oocytes and cleavage stage embryos, while fluorescein-labeled GST does not adopt a predominant nuclear location at these developmental stages. Panels A-E reflect oocytes and embryos co-injected with fluorescein-labeled GST-SP17 and Alexa594-labeled GST-NLS; panels F-G reflect oocytes and embryos co-injected with fluorescein-labeled GST-RREB and Alexa594-labeled GST-NLS; panels H-L reflect oocytes and embryos co-injected with fluorescein-labeled GST and Alexa594-labeled GST-NLS. In all cases panels beginning with the same letter are derived from the same representative oocyte or embryo; images were captured as an optical section using confocal microscopy. DNA is shown in panels A1–L1; fluorescein-labeled proteins are shown in panels A2–L2, Alexa594-labeled proteins are shown in panels A3–L3. A GV-stage oocyte (GV), pronuclear stage embryo (PN), 2-cell stage embryo (2-cell), 4-cell stage embryo (4-cell) and 8-cell stage embryo (8-cell) are shown as indicated in the figure.

To determine if the peptides identified by the LCMS analysis had the ability to be trafficked to the nucleus (and therefore likely cargoes for karyopherin α proteins), we produced GST-fusion versions of SP17 and RREB (proteins with putative nuclear localization signals that interacted with KPNA1 and KPNA7, respectively). As shown in Figure 2, both SP17 and RREB were able to adopt a nuclear localization in porcine oocytes and cleavage stage embryos. Both SP17 and RREB appeared to adopt a uniform distribution between the nuclear and cytoplasmic compartments in GV-stage oocytes when evaluated one hour following microinjection; the co-injected GST-NLS presented a predominantly nuclear localization (n=29, n=25 for SP17 and RREB, respectively). GST demonstrated a predominately cytoplasmic localization, while the co-injected GST-NLS presented a predominantly nuclear localization (n=28). In pronuclear stage embryos, both SP17 (n=8) and RREB (n=6) displayed a predominant localization in the pronuclei of all embryos, as did the co-injected GST-NLS control; GST protein was predominantly cytoplasmic in localization (n=7). SP17 adopted a strong nuclear localization in 2-cell (n=20), 4-cell (n=15) and 8-cell stage embryos (n=12) as did the co-injected GST-NLS; GST protein was predominantly cytoplasmic in localization in 2-cell (n=18), 4-cell (n=12) and 8-cell (n=11) stage embryos. All embryos injected with RREB arrested at the pronuclear stage of development.

### Time-dependent Nuclear Accumulation of SP17 and RREB in Porcine Oocytes

**Figure 3:**
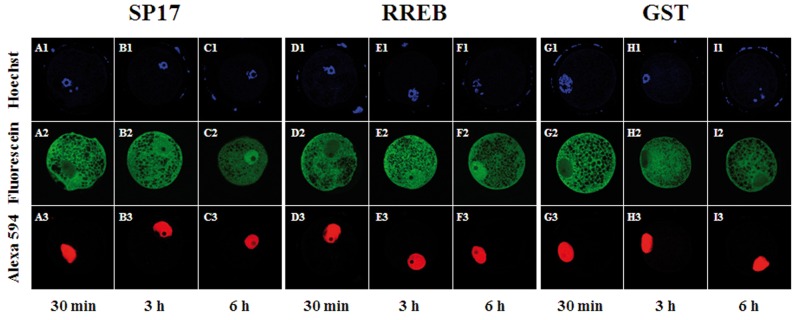
Nuclear accumulation of GST-SP17 and GST-RREB is time-dependent. Panels A-C reflect oocytes co-injected with fluorescein-labeled GST-SP17 and Alexa594-labeled GST-NLS; panels D-F reflect oocytes co-injected with fluorescein-labeled GST-RREB and Alexa594-labeled GST-NLS; panels G-I reflect oocytes co-injected with fluorescein-labeled GST and Alexa594-labeled GST-NLS. In all cases panels beginning with the same letter are derived from the same representative oocyte; images were captured as an optical section using confocal microscopy. DNA is shown in panels A1–I1; fluorescein-labeled proteins are shown in panels A2–I2, Alexa594-labeled proteins are shown in panels A3–I3. Both GST-SP17 and GST-RREB show a weak nuclear accumulation 30 minutes after microinjection into GV stage oocytes. GST-SP17 and GST-RREB both show an increased presence in the nucleus at 3 and 6 hours after microinjection. In contrast, the co-injected GST-NLS adopts a nuclear localization rapidly after microinjection and has a profound nuclear accumulation 30 minutes following microinjection. Fluorescein-labeled GST remains a predominantly cytoplasmic protein at all time-points following microinjection.

Because the nuclear accumulation of SP17 and RREB appeared lower in GV-stage oocytes, we assessed SP17 and RREB localization at 30 minutes, 3 hours and 6 hours after microinjection to determine if nuclear accumulation increased with time. As shown in Figure 3, both SP17 and RREB appeared to adopt a predominantly cytoplasmic localization 30 minutes following microinjection (n=25, n=21, respectively) while the co-injected GST-NLS adopted a predominantly nuclear localization. Three hours after microinjection both SP17 (n=23) and RREB (n=18) increased in nuclear abundance; in all cases GST-NLS possessed a predominant nuclear localization. Six hours after microinjection both SP17 (n=22) and RREB (n=15) possessed a strong nuclear localization, as did the co-injected GST-NLS. Fluorescein-labeled GST remained predominately cytoplasmic 30 minutes, 3 hours and 6 hours after microinjection, while the co-injected GST-NLS possessed a predominantly nuclear localization (n=20, n=17 and n=15, respectively).

### SP17 and RREB Interact with Karyopherin α Subtypes

**Figure 4:**
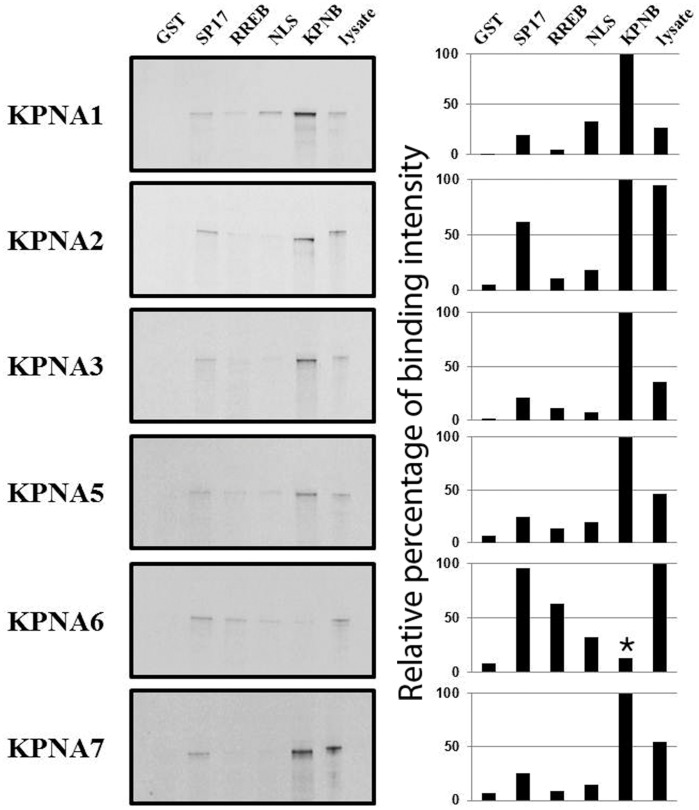
Recombinant GST-SP17 and GST-RREB interact with multiple karyopherin α subtypes. S^35^-labeled versions of porcine KPNA1, KPNA2, KPNA3, KPNA5, KPNA6, and KPNA7 were tested for their ability to interact with GST, GST-SP17, GST-RREB, GST-NLS, and a GST tagged version of karyopherin β (KPNB). Shown in this figure are the respective radiographs of this *in vitro* binding assay. Lanes are labeled as follows: GST (recombinant GST); SP17 (GST-SP17); RREB (GST-RREB); NLS (GST-NLS); KPNB (GST-KPNB), and lysate (crude lysate from the respective S^35^-labeled KPNA *in vitro* translation reactions, corresponding to 10% of input into each binding reaction). The intensity of the radiolabeled bands was quantified for each karyopherin α subtype; band intensity normalized to the intensity of binding to KPNB (100%) for KPNA1, KPNA2, KPNA3, KPNA5, and KPNA7. *Because the KPNA6 construct lacked the majority of the importin β binding domain, little interaction with KPNB was observed and in this case KPNA6 intensity was normalized to lysate input, rather than KPNB binding.

A series of *in vitro* binding assays were carried out to determine if the nuclear accumulation of SP17 and RREB was a result of their interactions with karyopherin α subtypes. As shown in the representative images in Figure 4, GST does not effectively interact with any karyopherin α subtype; all karyopherin α subtypes interact with either karyopherin β or GST-NLS. SP17 and RREB both interact with several karyopherin α subtypes. While all KPNA subtypes interact with SP17, KPNA2 displays the strongest interaction with SP17. RREB interacts weakly with all subtypes in a manner similar to GST-NLS; the interaction of RREB with KPNA1 and KPNA2 is markedly less robust than the interactions of SP17 with these karyopherin α subtypes.

## Discussion

The aim of this set of experiments was to determine if karyopherin α cargoes could be identified in porcine tissue using LCMS analysis of an *in vitro* pull-down assay. The goal of the current study was not to perform an exhaustive screen of all protein interactions between karyopherin α subtypes and the prey protein, nor was our objective to determine proteins that serve as endogenous cargoes for a given karyopherin α subtype in porcine oocytes and embryos. We chose to validate the results of the LCMS analysis by injecting recombinant, fluorescently labeled GST-SP17 and GST-RREB into porcine oocytes and embryos because it has been previously shown that KPNA1 and KPNA7 are expressed during this time in development [11]. Cloning a region of the open reading frame that contained a presumptive NLS in both SP17 and RREB was straightforward as both SP17 and RREB were predicted to have a classical NLS (Table 1). Because these presumptive cargoes could interact with recombinant KPNA1 and KPNA7 in an *in vitro* GST pull-down assay, we expected they would also function as substrates for nuclear import, regardless of whether or not the endogenous versions of SP17 and RREB were expressed in porcine oocytes and embryos, as shown in Figures 2 and 3.

While recombinant SP17 and RREB appeared to adopt a clear nuclear localization in porcine oocytes and embryos, the kinetics of the import of these two proteins differed dramatically from that of the GST-NLS protein (as shown in Figure 3) in porcine oocytes. Although the size of these proteins (SP17 and RREB fusion proteins being approximately 150–200 amino acids longer than the GST-NLS control), may be a factor in this apparent delayed accumulation of protein in the nucleus, studies have shown that the *in vitro* and *in vivo* transport kinetics of proteins of this size do not differ dramatically from those observed for GST-NLS [20]. Our study did not address the issue of rate of transport in embryos because we injected recombinant proteins at the pronuclear stage and collected embryos at discrete time points following fertilization. In this way, sufficient time likely passed for nuclear envelope formation after mitosis and the subsequent nuclear import of the labeled proteins, thereby precluding the possibility of assessing the rate that RREB or SP17 trafficked to the nucleus as compared to GST-NLS.

Despite the fact that we used only KPNA1 and KPNA7 as the bait proteins in our pull-down assay, it is certainly possible that the cargoes we identified also interact with additional karyopherin α subtypes. To test this possibility, we performed an *in vitro* binding assay to assess how well recombinant RREB and SP17 interacted with a series of *in vitro* translated karyopherin α subtypes. The results of this assay are outlined in Figure 4. Overall RREB and SP17 appear to be able to interact with KPNA1, KPNA2, KPNA3, KPNA5, KNPA6, and KPNA7. Interestingly, SP17 tended to show a stronger interaction with the above-mentioned karyopherin α subtypes than RREB. It is important to be cautious in over-interpreting the results from this assay. The results from this *in vitro* binding assay indicate RREB and SP17 can interact with multiple karyopherin α subtypes, but one must keep in mind these are *in vitro* conditions and that even though these proteins show an interaction *in vitro*, an *in vivo* interaction may not exist, perhaps because a given karyopherin α subtype is not present in a given cell type, or a given karyopherin α subtype is restricted in its ability to translocate to the nucleus. For instance, KPNA2 transcripts are in lower abundance in GV-stage porcine oocytes than transcripts for KPNA1, KPNA3, KNPA6, and KPNA7 [11]. It is unclear at what level endogenous KPNA2 protein exists as compared to the other KPNA subtypes in porcine oocytes, but a low level of KPNA2 could contribute to a low rate of KPNA2-specific cargo import.

It is interesting that injection of recombinant RREB led to developmental arrest in pronuclear stage embryos. While we have no direct evidence to explain this phenotype, it is likely that this ectopic protein contained a non-specific contaminant that was embryo toxic. Alternatively, nuclear accumulation of RREB may have interfered with critical cellular processes at the pronuclear stage of development (such as DNA replication or progression through mitosis). In any case, the goal of this assay was to determine if RREB was a cargo for nuclear import; further studies can be designed to determine the reasons behind why ectopic expression of RREB led to developmental arrest.

Because KPNA7 is an oocyte- and cleavage stage embryo-specific KPNA subtype, using a prey protein derived from cultured porcine fibroblast cells likely precluded our ability to identify proteins that are trafficked solely by KPNA7. However, as it is known that specific NLS-bearing proteins can interact with multiple karyopherin α subtypes, our findings are likely reflective of the fact that KPNA7 can bind and traffic proteins that are also transported by other members of the karyopherin α subfamily [13]. Using prey protein derived from oocytes (a cell type in which KPNA7 is expressed at significantly higher levels than the other karyopherin α subtypes) would enable us to screen a prey protein source that is likely enriched in KPNA7-specific cargoes. Identifying KNPA7 specific cargoes is a logical step toward understanding the developmental arrest phenotype observed in embryos in which KPNA7 is reduced by RNAi [9], [11]. Furthermore, identification of the cargoes trafficked by multiple members of the KPNA subfamily will facilitate an understanding of the differential phenotypes seen between the various karyopherin α subtype specific knockouts in mice [18], .

Three major factors likely contributed to the small number of proteins identified by our GST pull-down and LCMS screening approach. First, instead of subjecting the entire elution from the glutathione-agarose matrix to LCMS, we subjected only a single silver stained protein band excised from a polyacrylamide gel to LCMS. While this band certainly contained several proteins of similar molecular weight, this band likely represented less than 5% of the total protein eluted from the matrix. Secondly, the mass spectra that were recognized by our LCMS approach were mapped to the porcine protein database (NCBI); because the porcine genome is only in its early draft stages (version two at the time of our analysis), additional peptides could be identified from our peptide spectra as the annotation of the protein database advances. Lastly, because the LCMS approach we employed analyzes dominant peptides in real-time, peptides that are of minor abundance and elute at a time-point near that of a major peptide, peptides in minor abundance are not sequenced and are therefore lost in the analysis.

Certainly the LCMS approach employed in this set of experiments revealed some likely contaminant peptides and proteins that exhibited non-specific binding. For instance, both the KPNA1 and KNPA7 elution volumes contained trypsin, an expected result as all proteins were digested with trypsin prior to LCMS analysis. Keratin was also detected in both samples, likely due to aerosolized proteins from human skin introduced during the processing of the excised gel fragments. Aside from these proteins, not all proteins identified by our screen contained putative NLSs or have been reported to be present in the nucleus. For instance, the glutamate receptor ionotropic AMPA 1 precursor protein identified in the KPNA7 pull-down does not contain a putative NLS and its human ortholog has been shown to be a membrane bound protein [24]. While the presence of this protein in our elution could be due to a non-specific interaction, or simply an artifact of our *in vitro* GST pull-down assay, we did expect our assay to detect proteins that genuinely interact with karyopherin α subtypes that lack both a nuclear localization and an NLS. Proteins that are part of the cytoskeleton or nucleoporins that comprise the nuclear pore complex, for instance, likely interact with karyopherin α to mediate trafficking [25], although this interaction is likely not mediated by the NLS recognition portion of karyopherin α.

Other screening methods have been used to identify cohorts of KPNA subtype-specific binding partners. A yeast two-hybrid approach has been used to screen for potential cargoes of KPNA2 by using a cDNA library generated from murine testis cDNA [26]. This approach revealed the identity of three nuclear proteins that were previously unknown to play a role in spermatogenesis. In addition, a proteomic approach using 2D gel electrophoresis and matrix-assisted laser desorption ionization (MALDI) mass spectrometry has been used to identify proteins in the adult mouse brain that interact with importin α5 (KPNA1) [27], a karyopherin α subtype hypothesized to play a pivotal role in neuronal differentiation [20]. While both of these screening methods have yielded valuable insight into the binding partners of the murine KPNA2 and KNPA1 orthologs, we find our approach described herein a straightforward proteomic approach that can be used to identify heretofore unknown binding partners of the karyopherin α family of nuclear transport receptors.

In conclusion, we have shown a unique method to screen for intracellular binding partners for the karyopherin α family of transport receptors using a combination of a GST pull-down screen and LCMS proteomic analysis in a non-traditional model system. The fact that we were able to identify proteins that possessed NLSs and that we could validate a subset of these proteins as having both the ability to interact with karyopherin α subtypes (based on our *in vitro* binding assay) and be trafficked to the nucleus (based on our *in vivo* microinjection assay) supports the use of this approach to further screen additional prey protein extracts to identify karyopherin α interacting partners.
